# Harnessing the probiotic properties and immunomodulatory effects of fermented food-derived *Limosilactobacillus fermentum* strains: implications for environmental enteropathy

**DOI:** 10.3389/fnut.2023.1200926

**Published:** 2023-06-05

**Authors:** Vidhya Prakash, Aravind Madhavan, Archana Palillam Veedu, Pradeesh Babu, Abhirami Jothish, Sruthy S. Nair, Alin Suhail, Meera Prabhakar, Thasleema Sain, Raveena Rajan, Priyanka Somanathan, Kuniyil Abhinand, Bipin G. Nair, Sanjay Pal

**Affiliations:** School of Biotechnology, Amrita Vishwa Vidyapeetham, Kollam, Kerala, India

**Keywords:** probiotics, environmental enteropathy, *Caenorhabditis elegans*, fermented rice, *Limosilactobacillus fermentum*, HT 29, immunomodulation

## Abstract

**Introduction:**

Environmental enteropathy (EE), a chronic small intestine disease characterized by gut inflammation, is widely prevalent in low-income countries and is hypothesized to be caused by continuous exposure to fecal contamination. Targeted nutritional interventions using potential probiotic strains from fermented foods can be an effective strategy to inhibit enteric pathogens and prevent chronic gut inflammation.

**Methods:**

We isolated potential strains from fermented rice water and lemon pickle and investigated their cell surface properties, antagonistic properties, adhesion to HT-29 cells, and inhibition of pathogen adherence to HT-29 cells. Bacteriocin-like inhibitory substances (BLIS) were purified, and *in vivo*, survival studies in *Caenorhabditis elegans* infected with *Salmonella enterica* MW116733 were performed. We further checked the expression pattern of pro and anti-inflammatory cytokines (IL-6, IL8, and IL-10) in HT-29 cells supplemented with strains.

**Results:**

The strains isolated from rice water (RS) and lemon pickle (T1) were identified as *Limosilactobacillus fermentum* MN410703 and MN410702, respectively. Strains showed probiotic properties like tolerance to low pH (pH 3.0), bile salts up to 0.5%, simulated gastric juice at low pH, and binding to extracellular matrix molecules. Auto-aggregation of T1 was in the range of 85% and significantly co-aggregated with *Klebsiella pneumoniae, S. enterica,* and *Escherichia coli* at 48, 79, and 65%, respectively. Both strains had a higher binding affinity to gelatin and heparin compared to *Bacillus clausii*. Susceptibility to most aminoglycoside, cephalosporin, and macrolide classes of antibiotics was also observed. RS showed BLIS activity against *K. pneumoniae*, *S. aureus,* and *S. enterica at* 60, 48, and 30%, respectively, and the protective effects of BLIS from RS in the *C. elegans* infection model demonstrated a 70% survival rate of the worms infected with *S. enterica*. RS and T1 demonstrated binding efficiency to HT-29 cell lines in the 38–46% range, and both strains inhibited the adhesion of *E. coli* MDR and *S. enterica*. Upregulation of IL-6 and IL-10 and the downregulation of IL-8 were observed when HT-29 cells were treated with RS, indicating the immunomodulatory effects of the strain.

**Discussion:**

The potential strains identified could effectively inhibit enteric pathogens and prevent environmental enteropathy.

## 1. Introduction

Environmental enteropathy (EE), a condition of intestinal inflammation, is triggered by poor sanitation and frequent exposure to fecally contaminated food. It has been identified as the major reason for the growth stunting in children in developing countries (1). Applications of probiotic formulations to tackle EE have been investigated recently, and fermented foods harbor multitudes of these protective cultures. Fermented foods and drinks have been the most explored source for isolating beneficial (probiotic) bacteria that effectively control gut infections (2). Probiotic bacteria are live microorganisms that benefit human health when ingested in sufficient quantities (3). Improved intestinal microbial balance and immunomodulation, promotion of healthy gut microflora, inhibition of proliferation of harmful bacteria in the gastrointestinal barrier (4, 5), and offering new dietary alternatives for stabilizing the intestinal microflora are well-established attributes of probiotic strains (6).

Para probiotics, which employ inactivated probiotic bacteria, and synbiotics, which involve a combination of prebiotic components and probiotic bacteria, have also caught significant interest recently (7). Recent reports have shown that a diet rich in fermented foods could alleviate the microbiome diversity and concomitantly decrease inflammatory markers compared to a high-fiber diet, which alters the microbiome function by producing short-chain fatty acids and maintaining baseline microbial diversity (8). Distinct microbial populations are found in fermented foods, and these ingested food-derived microorganisms may display beneficial interactions with the existing gut microbiome. Metagenomic approaches could decipher the colonization impacts of these strains in the gut. Deviation in gut microbiota is associated with the enhanced risk of specific infections, and hence modulation of an unbalanced indigenous microbiota forms the rationale of probiotic therapy. Immunostimulatory and immunomodulatory effects of the probiotic strains have been studied extensively, and thus a combination of prebiotic, synbiotic, para-probiotic, and post-biotic approaches could be a better alternative in preventing microbial infections to a greater extent (9).

Cereal-based fermented food may contain multitudes of beneficial traits which can control metabolic disorders, including diabetes and hypercholesterolemia, and reduce cardiovascular diseases on account of their potent antioxidant properties and substantial vitamin and fiber content (10, 11). Fermented rice water and pickles are extensively used in traditional foods and beverages across the globe. Rice water, a starch suspension retrieved after boiling rice, has been widely used for decades to treat diarrhea induced by gastrointestinal diseases and cholera (12). They are rich sources of beneficial microbes (13). Pickles, too, when part of the diet in restricted quantities, have imparted similar effects (14).

Studies are now focusing on better understanding the immunomodulatory properties of probiotics, which may prevent or treat several illnesses which lack effective conventional treatment. Numerous studies have elucidated molecular mechanisms by which probiotics regulate intestinal epithelial health. Increased IgA secretion, cytokine production, production of antibacterial substances, improved intestinal barrier tight junctions against intercellular bacterial invasion, and competition with new pathogenic microorganisms for enterocyte adherence are predominantly significant (15). Hence, we isolated several strains from fermented food sources and performed *in vitro* investigations to evaluate their potential probiotic properties, such as host matrix binding, tolerance to low pH, bile salts and gastric juices, aggregation properties, and antipathogenic activities *in vitro* and *in vivo*. We also intended to investigate their adhesion ability to HT-29 cell lines, deciphered their antiadhesion potential against *E. coli* MDR and *S. enterica* MW116733, and performed the cytokine profiling of the strains to confer their immunomodulatory properties.

## 2. Materials and methods

### 2.1. Media, chemicals, instrumentation, and microbial strains

Lemon pickle was obtained locally (Amritapuri, Kollam, Kerala, India), and rice water was aseptically fermented for 6 h. De Man, Rogosa, Sharpe agar (MRS agar), and Nutrient Gelatin were obtained from HiMedia, India, to isolate *Lactobacillus* strains and gelatinase activity, respectively. For matrix binding studies, gelatin was obtained from HiMedia, India, and Heparin from Biological E. Limited, Hyderabad, India. Chromocult® Coliform Agar was purchased from Merck, India. Bile salts, Pepsin, and Ortho-nitrophenyl **–**D-galactopyranoside discs (ONPG) discs were procured from HiMedia, India. Solvents, hexadecane, and xylene were procured from Sigma-Aldrich, India. Dialysis tubing (3.5 K MWCO) was purchased from Thermo Fisher Scientific, India. *Bacillus clausii* spore suspensions 4 poly antibiotic-resistant *Bacillus clausii* (strains: O/C, N/R, SIN, and T) (Enterogermina®), *Klebsiella pneumoniae* (MTCC 3384) from Microbial Type Culture Collection, Chandigarh, India, *S.enterica* MW116733, *E. coli* MDR (Multidrug-resistant), *E.coli* ET (enterotoxigenic), and *Staphylococcus aureus* are clinical strains gifted by Dr. Bhabatosh Das, THSTI, Faridabad, India. HT-29 colon cancer cells were purchased from the National Centre for Cell Science, Pune, India. Cell lines were maintained in DMEM supplemented with 10% FBS, penicillin (100 U/ mL), and streptomycin (100 μg /mL) and maintained at 5% CO_2_ at 37°C.

### 2.2. Isolation of probiotic strains from rice water and lemon pickle

Locally available lemon pickle and fermented rice water were chosen to isolate the *Lactobacillus* strains in MRS agar at 37° C by spread plate method. The rice water sampled from cooked rice was aseptically fermented for 6 h and serially diluted up to 10^5^. In both cases, a few randomly selected colonies were further screened on their binding to gelatin. Gelatin coating was performed in flat bottom 96 well plates by incubating 100 μL of 10 mg/mL gelatin in each well at room temperature for 1 h, followed by rinsing with phosphate buffer saline (PBS) thrice. Bacterial colonies which grew on MRS agar was suspended in PBS and added to each well and maintained at room temperature. After 10 min, the bacterial suspension was discarded and washed with 100 μL PBS 5 times, and the fifth wash was spread plated. More stringent washes were carried out with 1 N NaCl in PBS followed by 1% dimethyl sulphoxide (DMSO) in PBS. The uniform small pinheaded colonies obtained from 1% DMSO-PBS fifth wash, considered a strong binder to the gelatin, was selected and subcultured for characterization. Rice water isolate was termed RS, and pickle isolates as T1 (16) (Supplementary Table S1).

### 2.3. Genomic DNA isolation and 16SrRNA sequencing for identification of strains

Genomic DNA isolation was performed by the phenol-chloroform method (14, 15). The DNA isolated was subjected to normal PCR with the help of 16 s rRNA gene primers forward primer (5’-AGAGTTTGATCCTGGCTCAG-3′), reverse primer (5’ACGGCTACCTTGTTACGACTT-3′) producing an amplicon of length 1.5 kb. After sequencing, BLAST analysis was performed (17).

### 2.4. Evolutionary analysis

BLASTn analysis of the sequences was done with the default parameters. The results obtained for aligned fasta sequences were downloaded from the NCBI BLAST, and phylogenetic analysis was carried out with the help of MEGA-X version 10.0.5. Fasta files were imported and aligned using MUSCLE and the resulting meg files were used to construct the phylogenetic tree using the Neighbor-Joining method with default parameters (18, 19).

### 2.5. Assessment of probiotic properties of the strains

#### 2.5.1. Acid tolerance ability

To determine the acid tolerance of the strains, overnight cultures of the strains were inoculated into tubes of MRS broth previously adjusted to pH values (1.5 and 3.0) with 1 N HCl and 1 N NaOH. The cultures adjusted to 0.1 optical density (OD) (following McFarland Standard) were inoculated, and aliquots of cultures exposed to pH 1.5 and 3.0 at 0 h and 3 h were plated onto MRS agar, and viable counts were determined. MRS broth maintained at pH 7.0 was used as the control (20). Experiments were performed in triplicates, plotting the average of 3 independent values.

#### 2.5.2. Bile tolerance ability

The bile salt tolerance was checked in MRS agar incorporated with bile salts in 0.2 and 0.5% concentrations. Aliquots of overnight cultures (100 μL) were spread plated onto the surface of the bile-salt-containing MRS agar, and viable counts were determined after 3 h of exposure. MRS broth without bile salts was used as a control (21).

#### 2.5.3. Tolerance to simulated gastric juice

The strains were centrifuged and resuspended in saline equivalent to an absorbance value of 0.05 OD at 600 nm. The cultures were then inoculated to simulated gastric juice (125 mM NaCl, 17 mM KCl, 45 mM NaHCO_3_, 3 g Pepsin) and adjusted to 3 different pH ranges 2.0, 3.0, and 7.0. Absorbance values after exposure to 6 h were measured at 600 nm (22).

### 2.6. Elucidation of cell surface properties

#### 2.6.1. Microbial adhesion to hydrocarbon test (MATH) assay for hydrophobicity

Bacterial cell surface hydrophobicity was assessed by measuring adhesion to hydrocarbons, hexadecane, and xylene. Overnight cultures of RS and T1 were centrifuged at 9,000 g for 10 min at 4°C. The pellet was washed with phosphate urea magnesium buffer (PUM buffer-22.3 g K_2_HPO_4_, 7.26 g KH_2_PO_4_, 1.80 g urea, 0.2 g MgSO_4_.H_2_O, pH 7.1). Absorbance at 600 nm was adjusted to 1 OD. To 5 mL cell suspension, 1 mL hexadecane was added. The two-phase system was vortexed for 2 min and incubated at 37°C for 1 h. The suspension was vortexed for 3 min and then incubated at room temperature for 1 h. The aqueous phase was removed, and absorbance was measured at 600 nm. Similarly, a two-phase system with xylene was performed. The percentage of cell surface hydrophobicity was expressed as.


$$\left(H\%\right)=\left(1-A1/A0\right)\times 100.$$

where A1 represents the absorbance of the aqueous phase after 1 h and A0 at time *t* = 0. *Bacillus clausii* was used as the positive control (23).

#### 2.6.2. Autoaggregation of probiotic strains

The strains (RS and T1) were grown in MRS broth and incubated at 37°C. The overnight cultures were centrifuged (5,000 g, 15 min, 4°C), harvested cells were washed twice with phosphate buffer saline (PBS), and resuspended in the same to a volume of 4 mL maintained at an OD of 0.2 (10^8^ CFU/mL). The cells were vortexed for 10 s, and the autoaggregation was determined at 3, 5, and 24 h, respectively. The top portion of the suspension was transferred to another tube with 3.9 mL of PBS, and the absorbance at 600 nm was measured each hour. The autoaggregation percentage is expressed as.


$$\mathrm{Autoaggregation}\;\left(\%\right)=\left[1\;-At/A0\right]\times 100.$$

where At denotes the absorbance at time *t* = 1–5 h and 24 h and A0 the absorbance at *t* = 0. *Bacillus clausii* was used as the positive control (24).

#### 2.6.3. Coaggregation of probiotic strains with pathogens

The coaggregation of probiotic strains with pathogens was investigated. The bacterial cell suspension was resuspended in PBS to approximately 10^8^ CFU/mL, and 2 mL of each bacterial suspension was mixed with 2 mL of RS and T1 each and vortexed for 10 s. (*S. enterica*, *E. coli* ET, *K. pneumoniae*). Each control tube with 4 mL of bacterial suspension alone was maintained at room temperature. The degree of coaggregation was measured at 600 nm for 0 h, 5 h, and 24 h, respectively, with the equation.


$$\mathrm{Coaggregation}=\frac{\left(\frac{\mathrm{Ax}+Ay}{2}\right)-A\left(x+y\right)}{\mathrm{Ax}+\frac{Ay}{2}}\times 100$$

where *x* and *y* represent each of the two strains in the control tubes, and (*x* + *y*) represents the mixture (25).

#### 2.6.4. Biofilm formation of probiotic strains

Enhanced biofilm formation is considered to be an ideal property that a probiotic strain should possess to bind to the gut barrier. Biofilm quantification was performed by inoculating 2 mL of an overnight culture of RS and T1 into 6 well microtiter plates, with initial turbidity of 0.05 OD at 595 nm. MRS broth was employed as a control. Plates were left in a static condition for 48 h at 30°C. Biofilm formation was analyzed by standard crystal violet assay as described by Gómez et al. Quantification was made based on the equations, non-biofilm producers [OD ≤ ODC], weak biofilm producers [ODC < OD ≤ 2 x ODC], moderate biofilm producers [2 x ODC < OD ≤ 4x ODC], strong biofilm producers [4 x ODC < OD] where ODC (cut-off) is mean OD value of the control. *B. clausii* was kept as a positive control. Visualization of biofilm formation was validated using acridine orange and crystal violet staining (26).

#### 2.6.5. Production of exopolysaccharides (EPS)

Bound EPS (EPS-b) and EPS released (EPS-r) from the strain were extracted using the following protocols. To extract EPS-b, a 50 mL overnight culture of strain was centrifuged at 11000 g for 25 min at 4°C. The pellet was suspended in 5 mL 1 M NaCl, and EPS-b was subjected to dissociation by sonication (40 W, 3 min, 4°C). Samples were then centrifuged at 6000 g for 30 min at 4°C. EPS-b was precipitated by adding two volumes of cold ethanol and incubated at 4°C. After incubation, the samples were centrifuged at 6000 g for 30 min at 4°C and were resuspended in 2 mL distilled water and dialyzed against 5 L distilled water for two days. To extract EPS-r, 50 mL overnight cultures were centrifuged at 10,500 g for 25 min at 4°C. Supernatants were treated with trichloroacetic acid (20%) and incubated under gentle agitation. The precipitated proteins were removed by centrifugation at 20,000 g for 25 min at 4°C. The EPS was precipitated from supernatant by adding 100 mL cold ethanol and incubated overnight at 4°C. Following centrifugation (6,000 g for 30 min, 4°C), the pellet was resuspended in 2 mL distilled water, dialyzed and the total carbohydrates in extracted EPS were estimated by phenol sulphuric acid method with glucose as standard (27).

### 2.7. Bacterial attachment to immobilized extracellular matrix (ECM)

Adhesion ability to ECM components provides substantial evidence to confirm the affinity of the strains to bind to the gut epithelium. Gelatin and heparin were employed to determine the binding efficiency of the proteins (1 mg/mL, 50 μL) added to 96 well plates and incubated at room temperature for 1 h. Unbound proteins were removed by washing with PBS. The absorbance of the strains was adjusted to 0.1 (600 nm), and 50 μL of each bacterial suspension was transferred to the coated plates and incubated for 1 h, 3 h, and 5 h, respectively, at room temperature. After each desired time interval, the wells were washed with 1X PBS thrice, and 50 μL of formalin was added and kept undisturbed for 20 min to fix the bacteria. After PBS wash, crystal violet (0.1%, 50 μL) stain was added for 1 min. The excess stain was removed by washing, and the plates were dried, followed by adding 50 μL of acetic acid to solubilize the dye. Absorbance was measured at 595 nm in a microplate reader using Gen5.2.05 software. *Bacillus clausii* and *E. coli* ET strains were used as controls (28).

#### 2.7.1. Microscopic examination of binding efficiency of the strains (fluorescent staining and crystal violet staining)

Microscopic observation of the probiotic binding to ECM was performed with some modifications (29). Cultures RS and T1 in MRS broth and *E. coli* ET and *B. clausii* in nutrient broth adjusted to 0.1 OD were inoculated into sterile coverslips in 6 well microtiter plates and incubated for 5 h. The wells were washed with PBS to remove planktonic cells, and coverslips were transferred to glass slides and heat-fixed at 55°C for 20 min. Fixed slides were then kept overnight at 4°C. After air drying for 15 min, slides were stained with 0.02% acridine orange for 3 min in the dark, washed with distilled water, and air dried at room temperature for 15 min. The attached cells were imaged using a fluorescent microscope (Olympus IX71). Another set of matrix-bound coverslips was stained with crystal violet (0.1%) and observed under 100 X magnification (Olympus BX51).

### 2.8. Detection of β-galactosidase activity

Qualitative determination of the rate of lactose fermentation was done by β**-**galactosidase assay. A single colony of RS and T1 was applied to ortho-nitrophenyl β **–**D-galactopyranoside discs (ONPG) in MIC tubes followed by the addition of 100 μL of saline. The reduction of ONPG to ONP (ortho nitrophenol) was indicated by a color change to yellow at room temperature (30).

### 2.9. Detection of gelatinase activity

In order to assess the safety of the strains to be employed as probiotic supplements, gelatinase production of the strains was checked. 1 μL aliquots of the 24 h cultures were spotted on to the surface of nutrient gelatin plates. Plates were incubated for different time periods and temperature, 37°C (48 h), 42°C (48 h), 25°C (72 h), 10°C and 15°C (10 d). After incubation, the plates were maintained at 4°C for 2 h and gelatin hydrolysis was recorded as opaque halos around the colonies. *Serratia marcescens* was used as a positive control (31).

### 2.10. Determination of antibiotic susceptibility of the strains

The susceptibility of the strains (RS and T1) to antibiotics was evaluated by the antibiotic sensitivity test by conventional Kirby-Bauer disc diffusion assay [32] with 10 different antibiotics, amoxicillin, (10mcg) chloramphenicol (30mcg), penicillin G (2 U), gentamicin (10 mcg), cefoxitin (30mcg), streptomycin (10 mcg), ampicillin (10mcg), amikacin (30mcg), erythromycin (15mcg), tetracycline (30mcg) (HiMedia). Overnight cultures of strains with OD (600) of 1 were swabbed uniformly across MRS agar plates; the antibiotic discs were placed and incubated for 24 h at 37°C. The diameter (mm) of zones of inhibition was measured, and data were interpreted based on Clinical and Laboratory Standards Institute (CLSI) guidelines as sensitive/susceptible (S), intermediate (I), and resistant (R) (33).

### 2.11. BLIS extraction by pH mediated adsorption desorption method and determination of antipathogenic potential

Initially, heating the culture broth to 60°C was done to prevent the inactivation of bacteriocin by proteases present in the culture medium. Adsorption of bacteriocin to producer cells was facilitated by adjusting the pH to 6.0 using 1 M NaOH followed by steering it for 30 min at 4°C. The cells were harvested by centrifuging at 10,000 g for 25 min at 4°C and pellets were washed twice with sterile 0.1 M phosphate buffer (pH 6.5). Pellets resuspended in 100 mM NaCl, was adjusted to pH 2.0 (1 N HCl), and stirred for 12 h at 4°C. Centrifugation of cell suspension at 10,000 g for 25 min was done and supernatant dialyzed against distilled water at 4°C for 24 h (dialysis tubing 3.5 K MWCO, Thermo Fisher Scientific). The protein concentration was determined by Bradford assay. The dialyzed samples were tested against indicator pathogens *S. enterica, S. aureus*, and *K. pneumoniae* by microtiter inhibition assay (34).

### 2.12. *In vivo* screening of Bacteriocin like inhibitory substance (BLIS) with *C. elegans* as infection model

Following egg extraction, an appropriate number of eggs was transferred into microtiter wells. The eggs were suspended in M9 broth and the synchronized L1 larvae were grown with *E. coli* OP50 as the food source, and development stages were examined. In order to study the survival percentage of the worms by infecting them with *S. enterica* and treatment with BLIS, the following setup was established with appropriate control. In the L4 stage, the worms were exposed to the following treatments (a) *C. elegans* + *S. enterica* (b) *C. elegans* + *S. enterica* + BLIS from RS (c) *C. elegans*  + *S. enterica* + antibiotic (ciprofloxacin 20 μg/mL) (positive control) and (d) *C. elegans* + M9 buffer (control). The survival percentage of the worms treated with BLIS was compared with L4 worms in M9 broth, and the survival curves were plotted and compared (32).

### 2.13. Adhesion ability of the strains to HT-29 cell lines

The adhesion potential of the strains to HT-29 cells was performed as described by Hugo et al. Briefly, cell suspension in concentration of 1×10^5^ was suspended in 4 mL of complete DMEM medium and transferred to six-well tissue culture plates. The medium was changed every alternate day and grown to 90% confluence at 37°C in 5% CO_2_ incubator. The spent medium was removed 24 h before adhesion assay and replaced with DMEM lacking antibiotics and incubated for 1 h at 37°C. After subsequent washes with PBS (pH 7.4), test strains RS and T1 maintained at OD_600_ of 1 (10^8^ CFU/mL) were added to respective wells and further incubated at 37°C for 2 h in CO_2_ environment. The monolayers were washed with PBS to remove non-adhered bacteria and trypsinized to detach the cells to make a homogenous suspension. Cell suspensions after serial dilution were plated onto MRS agar, and colony counts were performed after 24 h of incubation. The adhesion percentage was calculated as follows:


%adhesion=A1/A0×100.

where A0 is the initial number of bacteria added and A1 is adhered bacterial count (35).

### 2.14. Inhibition of pathogen adherence to HT-29 cell lines

Pathogen inhibition assays in cell lines were performed with two assays: competition and pathogen replacement methods. *E. coli* MDR and *S. enterica* were the test pathogens employed. Cell line was maintained as previously described. In the competition assay, equal volumes of the probiotic strains (RS and T1) maintained at OD_600_ of 1 were mixed with the pathogens and seeded to the wells and incubated for 1 h at 37°C in 5% CO_2_. Unbound bacteria were further removed, washed with PBS, detached by trypsinization and pathogen counts were performed in selective media, EMB (Eosine methylene blue) agar for *E. coli* and SS agar (Salmonella Shigella agar) for *S. enterica.* In replacement assay, pathogenic strains were added to the wells seeded with HT-29 cells and preincubated for 1 h at 37°C in 5% CO_2._ Unbound bacteria were removed, and RS and T1 strains were added to the replicate wells, respectively. After 1 h incubation, the protocol described earlier was repeated and the pathogen counts were performed to check the pathogen replacement ability of the strains. HT-29 cells seeded with *E. coli* MDR or *S. enterica* was employed as a control in both assays (36).

### 2.15. Effects of strain supplementation on immunomodulatory gene expression in HT-29 cells using real-time PCR

The inflammatory cytokine expression profiles of HT-29 cells in the presence of RS was determined. Briefly, strain maintained at OD_600_ of 1 was incubated with the monolayers for a time period of 6 h. Following incubation, the total RNA was isolated from HT-29 cells (Treated with RS and untreated) using NucleoSpin RNA extraction kit (Macherey-Nagel, Germany) according to the manufacturer’s protocol. RNA (1 μg) was converted to cDNA using the cDNA synthesis kit (Origin Diagnostics and Research, India). The cDNA synthesized was used for qPCR using the Real-time PCR master mix (Origin Diagnostics and Research, India) on an iCycler iQ real-time PCR detection system (Bio-Rad, USA). Fold changes in gene expression levels were assessed and were normalized to human actin (37). The list of oligonucleotide primers (Eurofins, India) is represented in Supplementary Table S2.

### 2.16. Statistical analysis

Statistical analysis of data obtained was performed by conducting Two-way RM ANOVA, and values were expressed as mean ± SD (Standard deviation of the mean) values of 3 independent experiments using the software Graph Pad Prism 8.0.2. (GraphPad Software, Inc., San Diego, CA). Significance levels were at *p ≤ 0.05, **p ≤ 0.01, ***p ≤ 0.001 and ****p ≤ 0.0001.

## 3. Results

### 3.1. Isolation of probiotic strains and 16 s rRNA typing

The fermented rice water isolate (RS) and pickle isolate (T1) were identified as *Limosilactobacillus fermentum* strains based on their molecular characterization. The strains were deposited in GenBank under (GenBank accession nos.MN410703, MN410702) which are rice water isolate and pickle isolate, respectively.

### 3.2. Evolutionary analysis

The analysis was conducted with MEGA5 using the neighbor-joining method. The optimal tree with the sum of branch length = 0.15357618 is shown. This analysis involved 95 nucleotide sequences. The pairwise deletion option removed all ambiguous positions for each sequence pair, with 1,549 positions in the final dataset. Evolutionary analyses were conducted in MEGA X, and the top 10 hits are shown. The strain MN410703 (RS) was not closely related to any other strains as analyzed from the BLAST analysis, which makes it an interesting candidate to elucidate the complete genome sequence (Supplementary Figure S1A). We have carried out blastn with the query sequence, and the related species with more than 90% sequence similarity with the query sequence was chosen. The strain MN410702 (T1) demonstrated an evolutionary relationship with strain MK639007 (Supplementary Figure S1B).

### 3.3. Assessment of probiotic properties of the strains

#### 3.3.1. Acid tolerance ability

**Figure 1:**
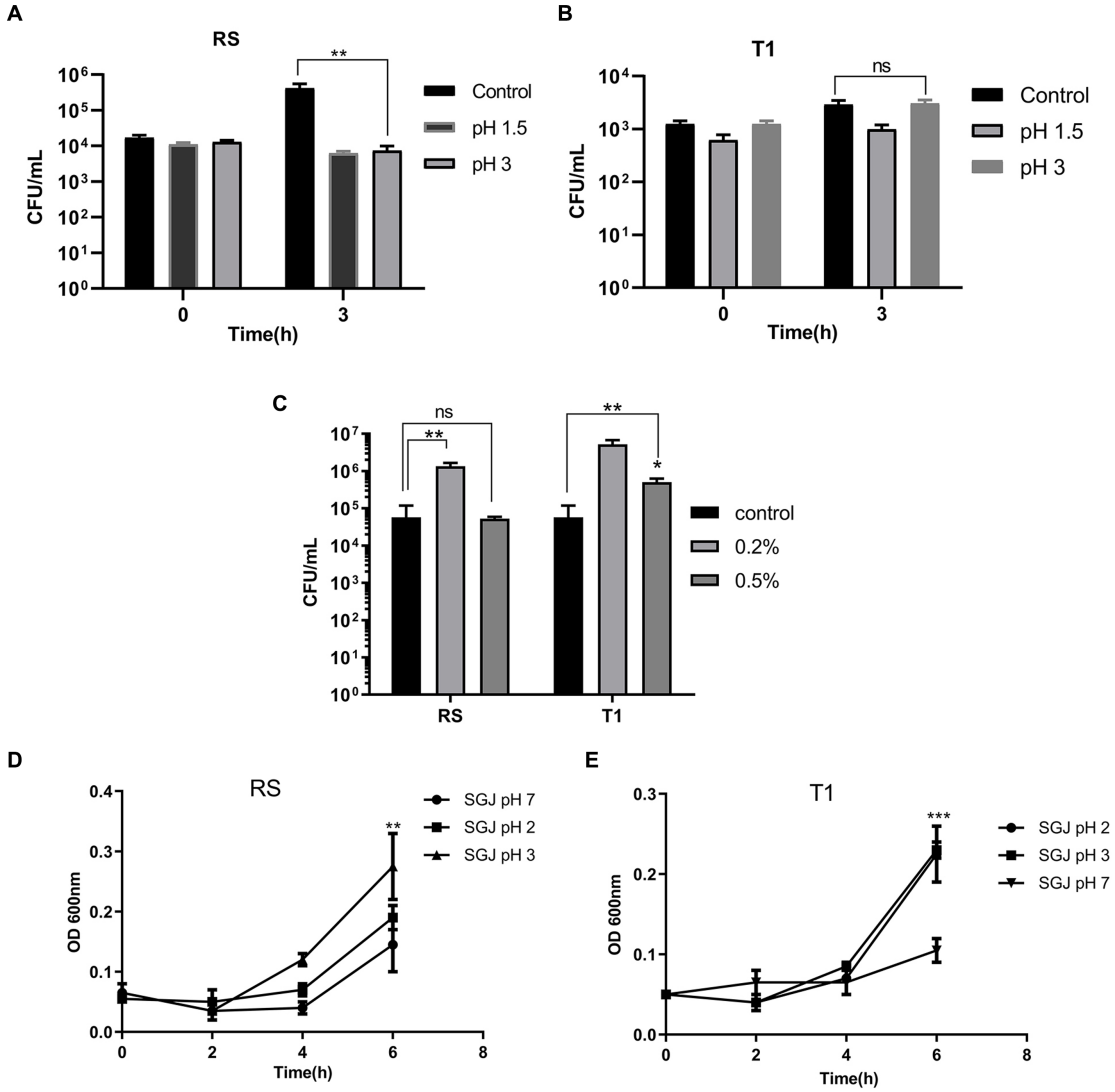
Probiotic properties of RS [*L. fermentum* (MN410703)] and T1 (*L. fermentum* MN410702). Viable counts of RS and T1 exposed to lower pH ranges after 3 h of exposure was measured (*p* ≤ 0.01) **(A,B)**. Viable counts of the strains after exposure to bile salts at 0.2 and 0.5% was determined (*p* ≤ 0.01) **(C)**. Absorbance values of RS and T1 after exposure to simulated gastric juice (SGJ) at pH 2, 3, and 7 was determined **(D,E)**. Values were expressed as mean ± SD of three individual experiments.

When compared to the control (pH 7.0) after exposure for 3 h, the colony counts of RS did not decrease in both pH 1.5 and 3.0, indicating their ability to persist and grow in the highly acidic environment (*p* ≤ 0.01) (Figure 1A). However, there was a negligible growth reduction when the organism was exposed to pH 1.5 for 3 h. T1, after 3 h of exposure, demonstrated increased growth at pH 3.0 compared to the control (pH 7.0) (Figure 1B).

#### 3.3.2. Bile tolerance ability

Viable counts of T1 and RS were determined after 3 h of exposure to 0.2 and 0.5% of bile salts. Results demonstrated that both RS significantly increased in counts by one log when exposed to bile salt concentrations of 0.2% (*p* ≤ 0.01) when compared with the control (without bile salts). T1 could tolerate 0.2% (*p* ≤ 0.01) and 0.5% (*p* ≤ 0.05) of bile salts, which was evident by the increase in CFU/mL (Figure 1C).

#### 3.3.3. Tolerance to simulated gastric juice

The absorbance values after 6 h of exposure demonstrated that with increasing time, T1 showed an increase in absorbance in simulated gastric juice (SGJ) at pH 2.0 and pH 3.0 (*p* ≤ 0.001) (Figure 1D). In contrast, RS showed increased absorbance at pH 3.0 compared to the control pH 7.0 (p ≤ 0.01), confirming the ability of the strains to tolerate the high acidity in the gastric environment (Figure 1E).

### 3.4. Elucidation of cell surface properties

#### 3.4.1. Microbial adhesion to hydrocarbon test (MATH) for hydrophobicity

**Figure 2:**
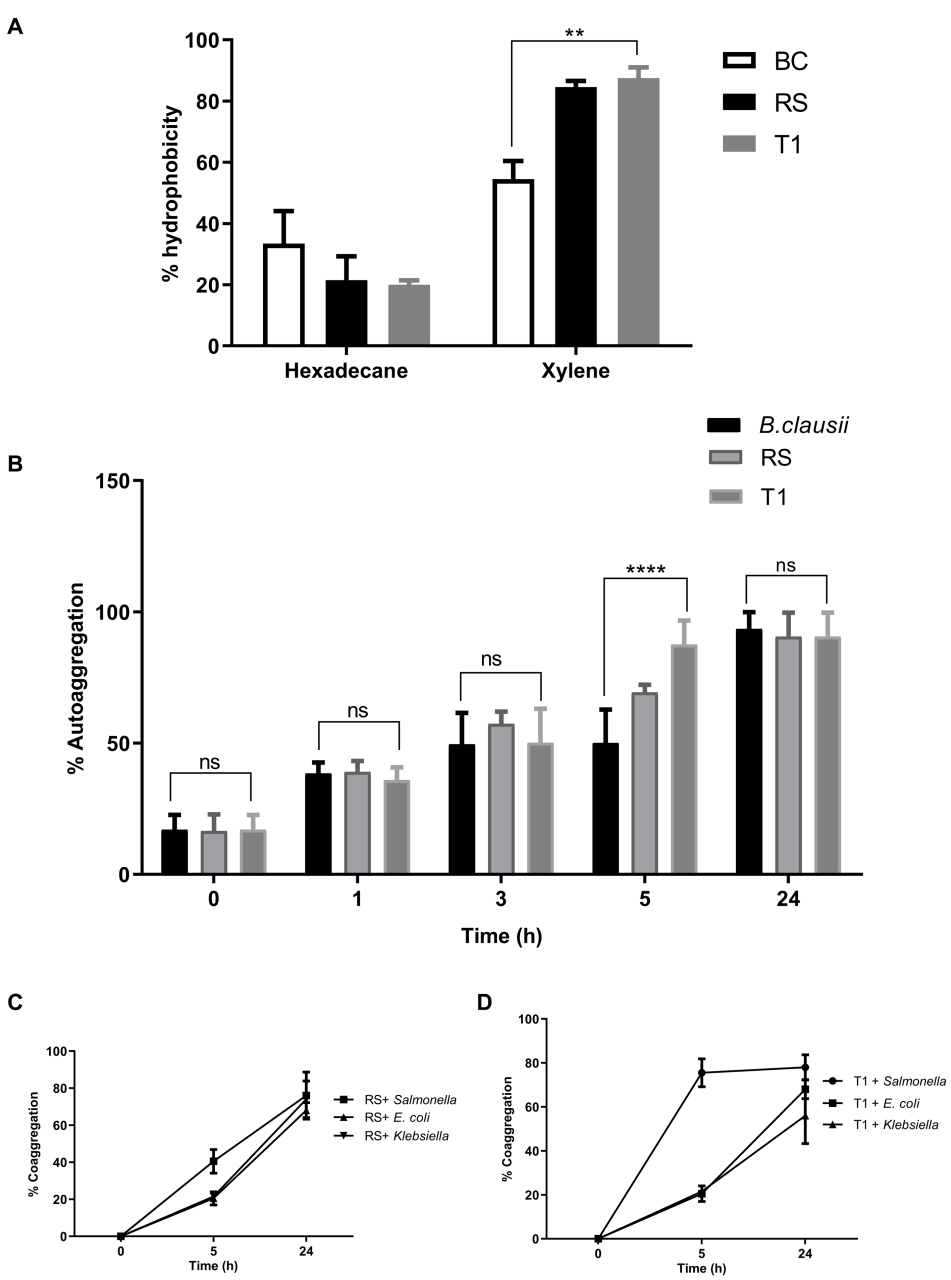
Elucidation of cell surface properties. Percentage of hydrophobicity exhibited by RS and T1 toward hexadecane and xylene was determined by MATH assay **(A)**. Auto aggregation percentages of RS and T1 until 24 h was compared with *B. clausii* and *E. coli* ET (*p* ≤ 0.001) **(B)**. Coaggregation percentages of RS and T1 strains with the pathogens, *S. enterica*, *E. coli* ET, and *K. pneumoniae* after 24 h of incubation **(C,D)**. Statistical Analysis of data obtained was performed by conducting two-way RM ANOVA.

The result indicated that compared to *Bacillus clausii*, RS and T1 had more affinity toward xylene. (*p* ≤ 0.01) with 88 and 90% for RS and T1, respectively. However, affinity to hexadecane was 25 and 21% (Figure 2A). The results confirmed the ability of strains to bind to hydrophobic barriers within the gut epithelium.

#### 3.4.2. Autoaggregation

The aggregation properties of the strains were compared with *B. clausii* and *E. coli* ET. After 5 h compared to *Bacillus clausii*, T1 exhibited a higher percentage of autoaggregation in the range of 85% (*p* ≤ 0.001). After 24 h of incubation, both strains exhibited 85% autoaggregation compared with the control (Figure 2B). This property will help the probiotic strains adhere to the gut epithelium and enhance their coaggregation abilities.

#### 3.4.3. Coaggregation

RS coaggregated with the pathogens in the range of 65% after 24 h (*p* ≤ 0.0001) (Figure 2C). In agreement with the autoaggregation results, T1 coaggregated with *S. enterica* in a range of 78% within 5 h of incubation. While with *E. coli* and *Klebsiella* spp. percentages were 65 and 45%, respectively, after 24 h of incubation (p ≤ 0.0001) (Figure 2D). High coaggregation percentages reveal the ability of the strains under investigation to competitively exclude pathogens from the gut epithelia, activating an immune response.

#### 3.4.4. Biofilm formation

**Figure 3:**
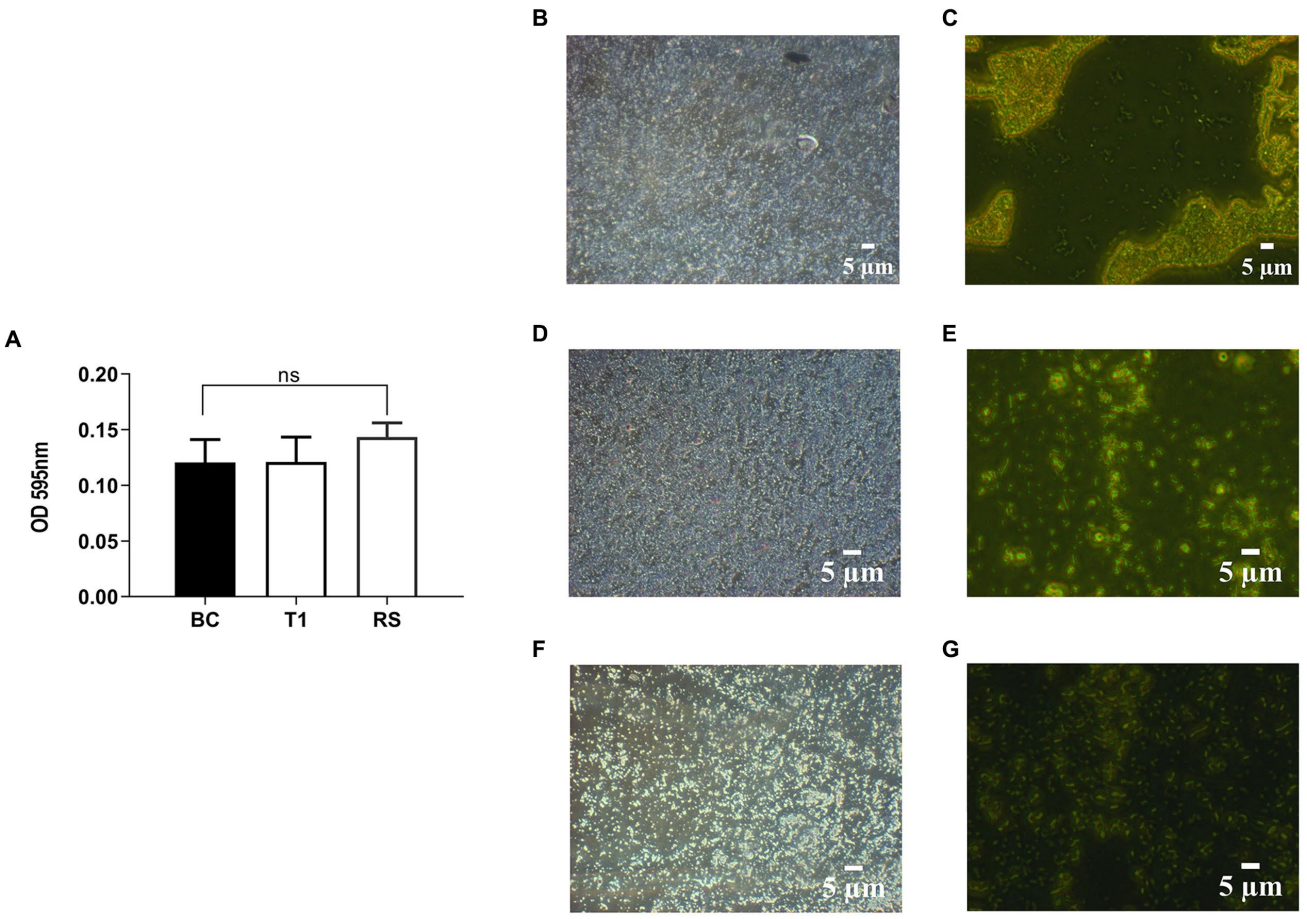
Quantitative analysis of biofilm formation was performed in comparison with *B. clausii* (BC) **(A)**. Biofilm formation ability of RS and T1 and crystal violet stained and acridine orange-stained images of BC **(B,C),** RS **(D,E)**, and T1 **(F,G)**. Values were expressed as mean ± SD of three individual experiments (two-way RM ANOVA).

RS and T1 were found to be moderate biofilm producers, as indicated by the absorbance values obtained compared with *B. clausii* (Figure 3A). Further validation of the observation was confirmed by CV staining and fluorescent microscopic observation (acridine orange) of the biofilms. Both RS (Figures 3D,E) and T1 (Figures 3F,G) formed biofilms abundantly on the surface indicated by the microscopy, which could strongly correlate with their aggregation, coaggregation abilities, and hydrophobicity percentages in comparison with *B. clausii* biofilms (Figures 3C,D).

#### 3.4.5. Production of exopolysaccharides (EPS)

EPS production of RS and T1 was quantified as 25 mg and 31 mg (dry weight) % with a total carbohydrate content of 52 mg/L and 58 mg/mL from the bound fraction. EPS-r fraction yields were less in both organisms. EPS production indicates the capability of the producer bacteria to deal with the harsh conditions of the upper part of the gut tract. However, productions seem to be both polymer and strain dependent. The physical and chemical properties of EPS is specific to each polymer type, which accounts for their applications in biological and industrial applications (38).

### 3.5. Bacterial attachment to immobilized extracellular matrix (ECM)

**Figure 4:**
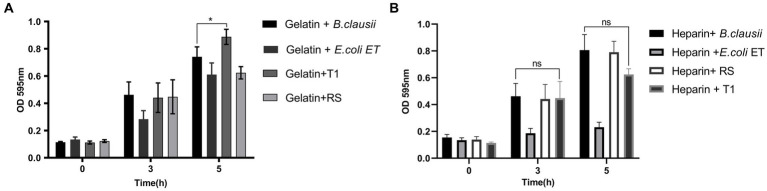
Bacterial attachment to immobilized extracellular matrix (ECM); Gelatin **(A)** (*p* ≤ 0.0001) and Heparin **(B)**. Binding efficiency was compared with *Bacillus clausii*. Statistical analysis of data obtained was performed by conducting two-way RM ANOVA.

**Figure 5:**
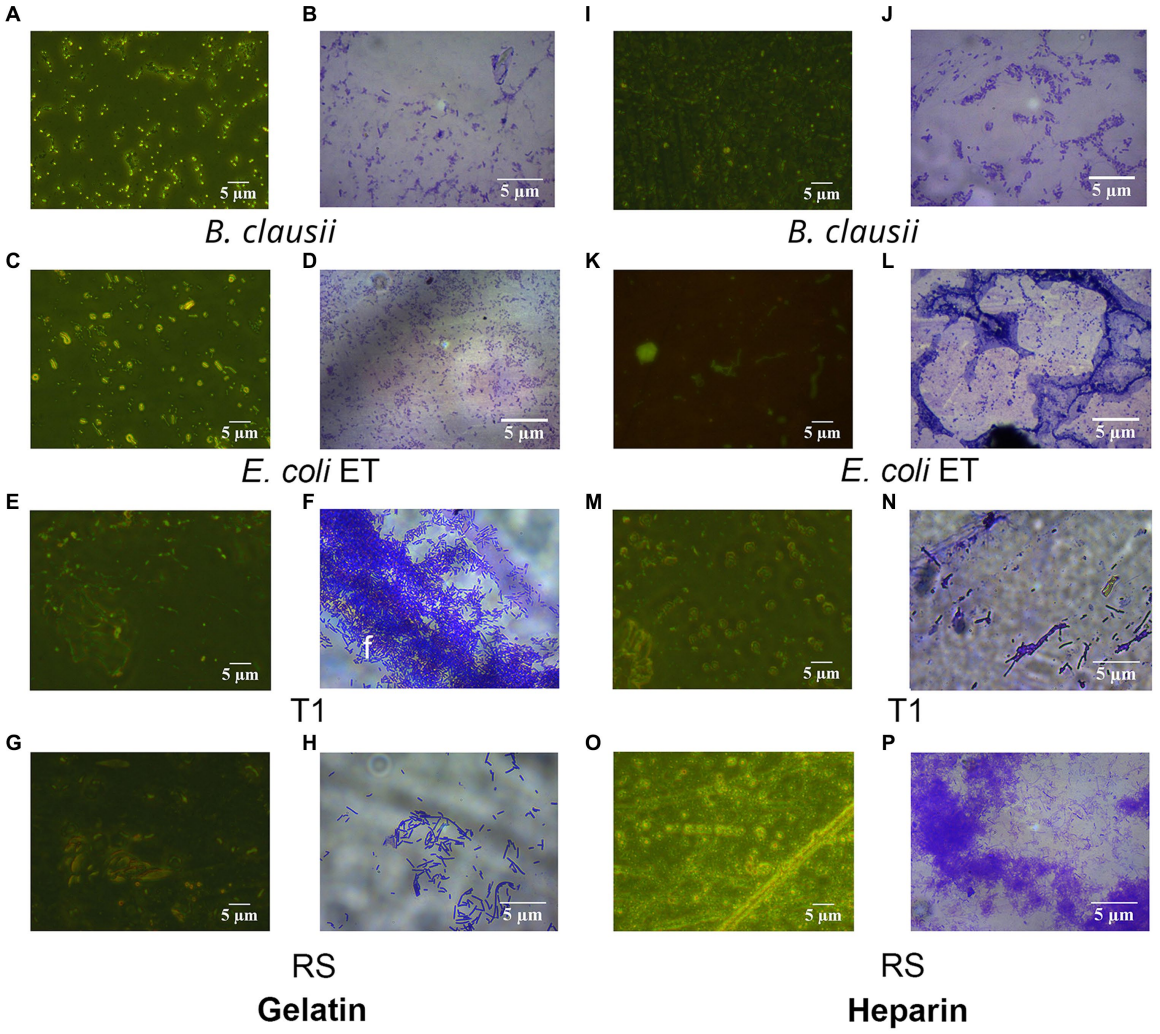
Fluorescent and Crystal violet-stained images of RS and T1 in gelatin and heparin matrix. Crystal violet (CV) stained and acridine orange-stained images of *B. clausii* **(A,B)**, *E. coli* ET **(C,D)**, T1 **(E,F)**, and RS **(G,H)** binding to gelatin. CV stained and acridine orange-stained images of *B. clausii* **(I,J)**, *E. coli* ET **(K,L)**, T1 **(M,N)**, and RS **(O,P)** binding to heparin. Microbial adherence after 5 h of binding to the matrix was recorded and compared with *B. clausii.*

To establish the strain’s ability to bind to the ECM, *in vitro* assays were performed, followed by microscopic observation. T1 demonstrated increased binding efficiency to the gelatin matrix until 5 h consistently when compared to *B. clausii* (*p* ≤ 0.001). The binding of RS to the gelatin matrix was similar to the pathogen model *E. coli* ET after 5 h (Figure 4A). RS and T1 bound with similar affinity as demonstrated as *B. clausii* with heparin (Figure 4B). It was observed that binding of *E. coli* was significantly reduced after 5 h of incubation. Results were confirmed by fluorescent microscopy and crystal violet staining. The strains were found to bind abundantly to the matrix after 5 h of incubation with gelatin (Figures 5A–H) and heparin (Figures 5I–P) when compared to *B. clausii* and *E. coli* ET, as revealed by microscopic observation.

### 3.6. Detection of β-galactosidase activity

β–galactosidase production of RS and T1 was confirmed qualitatively with ONPG discs, and within 24 h, yellow color development in MIC tubes indicated that the strains were early lactose fermenters (Supplementary Figure S2).

### 3.7. Detection of gelatinase activity

Both strains showed no gelatin hydrolysis zones after different incubation conditions indicating the absence of gelatinase, when compared to *Serratia marcescens* used as the positive control. The absence of gelatinase provided evidence of the non-virulent nature of the strains under investigation, which increases their modulation to be employed in a wide range of applications in food based applications (Supplementary Table S3).

### 3.8. Determination of antibiotic susceptibility of the strains

T1 demonstrated resistance to penicillin (beta-lactam), amikacin and streptomycin (aminoglycosides), and vancomycin (glycopeptide) but was sensitive toward all other antibiotics tested. RS strain was found resistant to vancomycin and amikacin but showed a sensitivity pattern to all other antibiotics tested, which would imply the absence of antibiotic-resistant genes being harbored, thereby promoting the safe probiotic applications of the strains (Supplementary Table S4).

### 3.9. BLIS extraction by pH mediated adsorption desorption method and determination of antipathogenic potential

**Figure 6:**
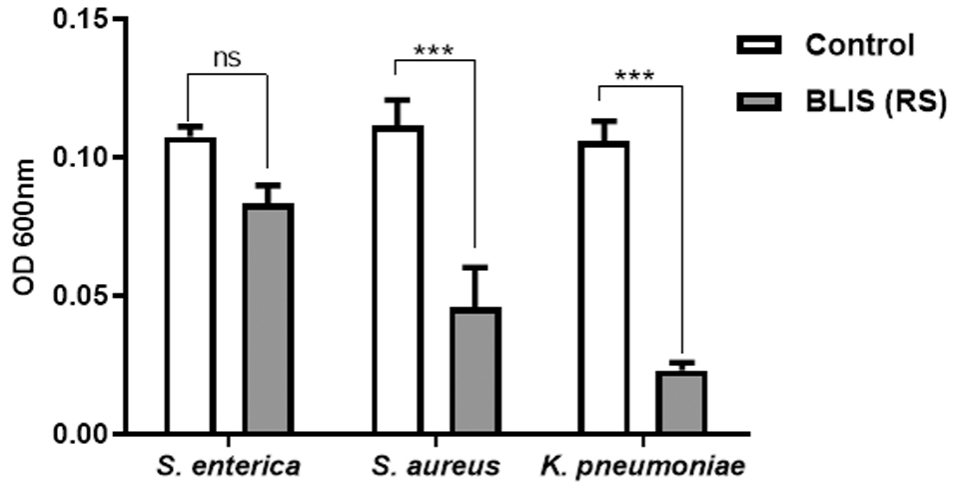
Inhibitory effects on pathogens treated with bacteriocin like inhibitory substance (BLIS) from RS (*p* ≤ 0.0001). Controls employed was untreated bacterial culture. Values were expressed as mean ± SD of three individual experiments (two-way RM ANOVA).

BLIS from RS in a concentration range of 0.5 mg/mL specifically demonstrated inhibitory activity against the pathogens *S. enterica*, *S. aureus,* and *K. pneumoniae,* with percentage inhibition rates of 30, 48, and 60%, respectively (*p* ≤ 0.001) (Figure 6). However, the effect of T1 was not prominent against the strains in the same concentration range.

### 3.10. *In vivo* screening of Bacteriocin-like inhibitory substance (BLIS) with *C. elegans* as infection model

**Figure 7:**
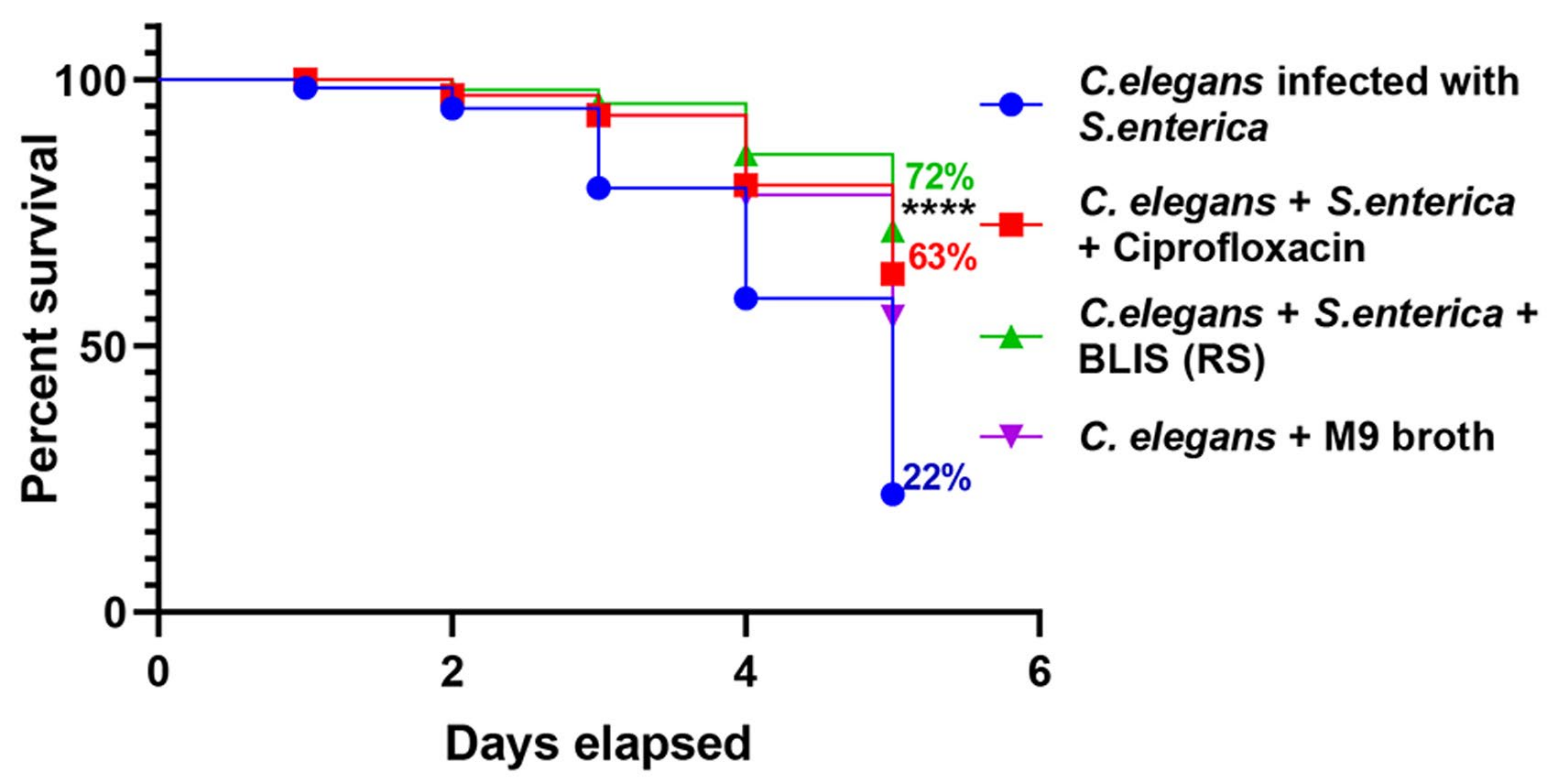
Effects of Bacteriocin like inhibitory substance (BLIS) from RS in *C. elegans* as infection model. Survival rate of the worms infected with *S. enterica* MW116733 was determined after treating with BLIS from RS strain. Worms treated with ciprofloxacin (20 μg/mL) was used as the control.

The survival rate of *C. elegans* infected with *S. enterica* after treatment with BLIS fraction of RS was determined and Kaplan–Meier survival curves were plotted. The survival rate of the pathogen-infected worms was around 22% after 5 days of infection. Results demonstrated that RS-BLIS-treated worms infected with *S. enterica* were found to be active after 5 days of treatment with a survival rate of 72% when compared with the worms treated with antibiotic ciprofloxacin (63%) (Figure 7). Results confirmed the potential of antimicrobial compounds from RS in inhibiting the growth of *S. enterica*. The results could infer that the antagonistic traits displayed against the pathogens may be attributed to the BLIS activity of RS in addition to several other metabolites, which could be deciphered in the future.

### 3.11. Adhesion ability of the strains to HT-29 cell lines

**Figure 8:**
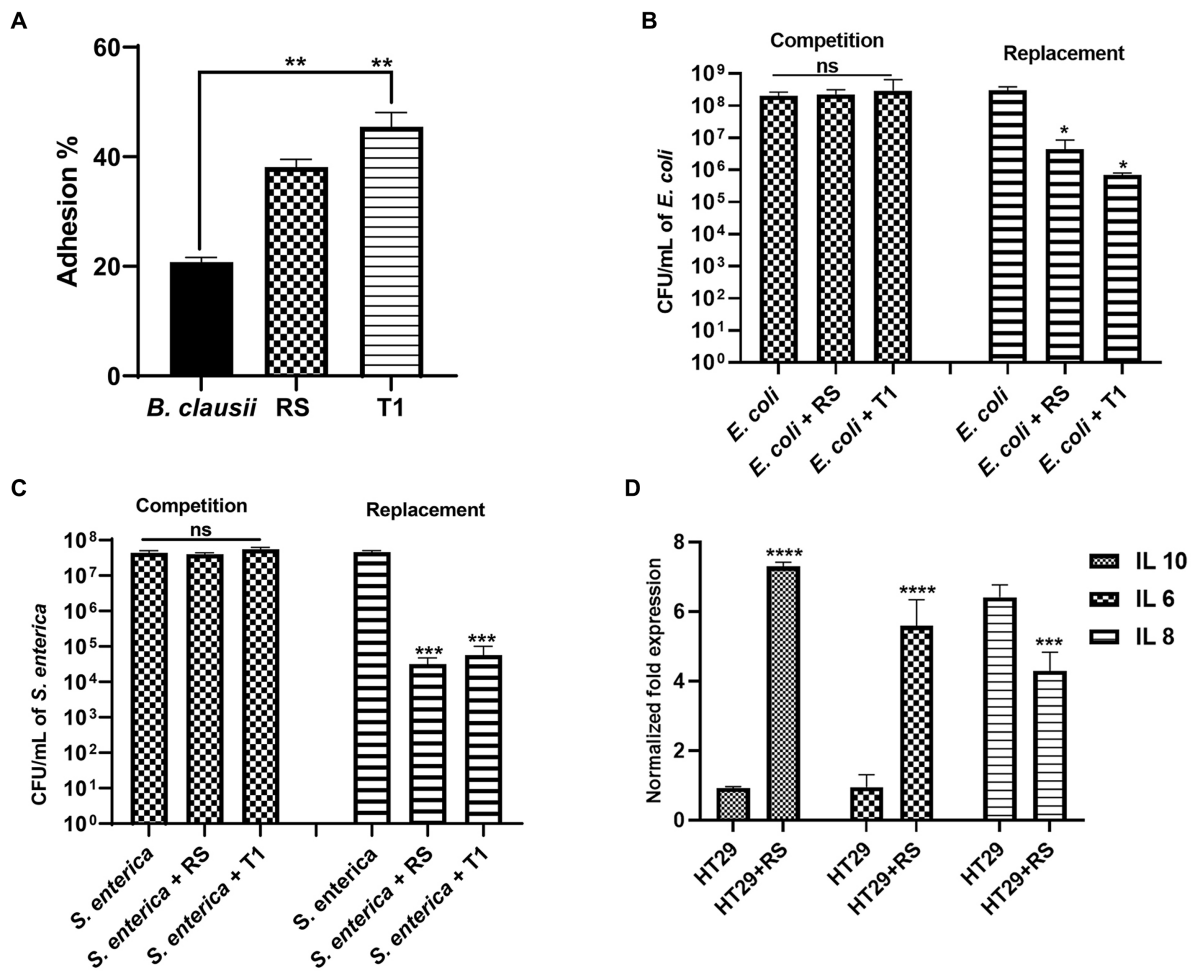
Adhesion potential of RS and T1 to HT-29 cell lines **(A)** (*p* ≤ 0.01). *B. clausii* was employed as a control. Inhibitory potential of RS and T1 against *E. coli* MDR in HT-29 cell lines (*p* ≤ 0.05) **(B)**. Inhibitory potential of RS and T1 against *S. enterica* in HT-29 cell lines (p ≤ 0.001) **(C)**. Elucidation of cytokine expression in HT-29 cells treated with RS **(D)**.

Both RS and T1 demonstrated autoaggregation properties and produced EPS in significant amounts. To further confirm their efficiency in binding to the intestinal epithelium, a prerequisite property that a prospective probiotic candidate should possess, we attempted to understand the adhesion ability of both strains to HT-29 cell lines. RS and T1 demonstrated adhesion percentages in the range of 38 and 46%, respectively, when compared with *B. clausii,* which was in the range of 22% (*p* ≤ 0.01) (Figure 8A). These observations could validate their colonization potential in complementing the strains’ cell surface properties demonstrated in the previous experiments.

### 3.12. Inhibition of pathogen adherence to HT-29 cell lines

We attempted to check the ability of the strains to compete with or replace pathogens (*E. coli* MDR and *S. enterica*) in HT-29 cell lines. Results suggested that RS and T1 demonstrated replacement ability against *E. coli* MDR, which was evidenced by 2 logs and 2.5 logs reduction in the colony forming units, respectively (*p* ≤ 0.05) (Figure 8B). Similarly, RS and T1 showed the replacement ability of *S. enterica,* evidenced by a 4-log reduction in colony counts of the pathogens (*p* ≤ 0.001) (Figure 8C). These observations substantially proved the ability of the strains to exclude and replace the pathogen load in the simulated gut environment. However, both strains did not show substantial competence in HT-29 cell line, as evidenced by the unaltered colony counts of the pathogens in the competition assay.

### 3.13. Effects of strain supplementation on immunomodulatory gene expression in HT-29 cells using real-time PCR

We investigated the impact of the *L. fermentum* (RS) on the human colon cancer cell line. More precisely, we analyzed the gene expression levels of the anti-inflammatory cytokines IL-6, IL-10, and pro-inflammatory cytokine IL-8 by qPCR upon treatment of HT-29 cells with RS. The results showed that treatment with *L. fermentum* induced upregulation of the expression of IL-6 and IL-10 (*p* ≤ 0.0001) and downregulated the expression of IL-8 at the RNA level (*p* ≤ 0.001) (Figure 8D). These results indicate that RS may enhance innate immunity by positively modulating anti and pro-inflammatory cytokines at the RNA level.

## 4. Discussion

The poor sanitation standards and fecal contamination often fuel environmental enteropathy, a major public health concern in developing countries. Earlier studies have targeted multiple approaches to tackle the EE-induced inflammatory responses, potentially looking at plant flavonoids, multivitamin supplementation, metal ions, and probiotics (39). The animal feed industry had employed piglet based *in vivo* studies in which weanling diets were involved in their growth promotion supplemented with probiotics, β-glucans, and plant extracts (40). However, human trials to employ probiotics as supplements for controlling EE are still under investigation (41). EE is often associated with dysbiosis in the gut microbiome, and associated profound inflammation stature could displace the niche of potentially protective bacteria in the gut. Dysbiosis could also be triggered by the prolonged use and synthesis of antibiotics in multiple communities and hospitals, which fuelled antimicrobial resistance (42). Recent studies implicate that the animal food sector accounts for over 70% of global antimicrobial applications (43). Sewage treatment plants are becoming the breeding ground of antimicrobial resistance (AMR), genes where sensitive strains freely mix with resistant ones derived largely from non-therapeutic sources, particularly food, and agriculture (44). It is high time we develop an integrated antimicrobial use policy emphasizing effective management of this health emergency. Moreover, the development of probiotics to alter microbial dynamics to favorable microbiomes is complex, as probiotic engineering by strains needs a more nuanced approach by analyzing the microbiome composition and the keystone species in that particular environment. In parallel, we need a traditional culture-based approach and trial and error methods for which one or few species must be experimentally tested for their potential to reduce the target pathogens in complex ecosystems such as food and broader industrial applications.

Previous reports have suggested that rice bran-based prebiotics in synergy with probiotics in the gut may promote gut health by producing metabolites (45). Fewer studies have investigated the potential probiotic strains from rice water fermented for 6 to 7 h. Several studies have claimed microbial diversity in different ethnic rice-based fermented foods but not in rice water which is widely drunk across rice cultivating regions worldwide, for its potential health benefits (46). Rice water with proper supplementation is also recognized as equivalent or better than WHO-formulated glucose-based ORS in many respects, as described by (12, 47). Very often, this boiled rice water gets fermented while stored. Understanding the possible microbes involved in this fermentation and their effects on human health is very important from a public health perspective, keeping in view of its wide usage in food, dermatological and industrial applications (48). However, applying these strains in the food sector demands identifying bacteria to the species level and validating their probiotic properties and safety guidelines.

An effective prerequisite property of probiotic strains is their ability to survive harsh conditions in the gastrointestinal tract (49). Therefore, the two strains, RS and T1, identified as *L. fermentum* MN410703 and MN410702, were tested for their acid and bile salts tolerance and survival ability in simulated gastric juice conditions. At pH 2.0, organisms could survive, but they grow better at pH 4.0, similar to observations made by (50). In contrast, viable counts indicated that strains could tolerate low pH ranges of 1.5 and 3.0. This is in agreement with studies done in probiotic strains isolated from cocoa fermentations (51). The resistance to low pH is an essential characteristic for the food industry as they withstand acidic environments for long periods.

Bile tolerance is crucial for the growth and survival of the strains in the proximal part of the small intestine. The liver synthesizes bile salts from cholesterol and is an essential candidate in the absorption and digestion of fats (52). Food stays in the small intestine for 4 to 6 h, and the mean bile concentration is 0.5% in the small intestine. Earlier investigations have proved that viability in 0.3% is considered optimum for bile-resistant strains (53). Our strains showed good tolerance after 3 h of exposure to 0.2 and 0.5% concentrations. The acidic gastric condition in the stomach destroys most of the microorganisms. RS and T1 survived in simulated gastric juice for 6 h and showed significant growth. Their significant transit tolerance was in full agreement with the similar tolerance of probiotic strains isolated from broiler chicken (52).

Hydrophobicity, a vital factor of a bacterial cell that shows the adhesive reaction to the intestinal surface, also enhances its tendency to form a biofilm (54). The investigations proved that our strains showed affinity to xylene in higher ranges when compared to hexadecane. Strains with more than 40% hydrophobicity percentages show that they are hydrophobic (55) and effectively colonize the intestinal walls. Autoaggregation can prevent or act as a barrier against the colonization of pathogens (56). The probiotic strain’s strong aggregating nature may help achieve an adequate mass to form biofilms (57). In this study, both strains showed good aggregation properties. T1 showed a consistent range of autoaggregation properties until 24 h. They were also strongly coaggregating with pathogens indicating their competitive exclusion properties. In alignment with aggregation properties, both strains were found to be moderate biofilm producers. The correlation between autoaggregation, coaggregation, and biofilm formation properties of the strains was in agreement with the observations by Vlková et al. (58). The EPS production by the strains confirmed the protective effects of the strains. EPS secreted by probiotic strains has been proven to modulate the immune system, promote anti-inflammatory activity and display antihyperalgesic activity in rats, as described by Dinic et al. (59).

Successful probiotic bacteria should be able to colonize the mucosal surfaces, at least temporarily, and prevent the attachment of pathogens such as *E. coli* and other intestinal or food-borne pathogens (60). The expression of cell surface adhesins which recognize and bind to ECM, play a vital role in preventing pathogen colonization and infection. It has been reported that exposed ECM, which is more susceptible to infection by the pathogen, is competitively blocked by probiotic strains by occupying their potential binding sites in the gut (61). Studies by Xueyan et al. have indicated that S-layer protein from *L. crispatus* interacts directly with collagen molecules on epithelial cells resulting in the competitive exclusion of gut pathogens (62). Our *in vitro* studies with gelatin and heparin have proved the affinity of the strains to bind matrix molecules more efficiently when compared to *B. clausii,* suggesting their potent ability to dominate pathogen binding. More investigations on cell adhesion molecule involved in binding has to be elucidated. We could further look at the characterisation of Surface layer proteins (SLPs) which can be a predominant factor in the adhesion mechanism of the strains.

Production of β-galactosidase, an industrially important enzyme, is a characteristic of *Lactobacillus* strains. The addition of lactobacilli-producing β-galactosidase as a probiotic in milk and cheese helps alleviate lactose intolerance symptoms (63), and the ability of our strain to produce this enzyme will enhance their beneficial effects. The absence of gelatinase and metalloproteinases (MMPs) secreted by pathogenic bacteria provided evidence of the non-virulent nature of the strains under investigation. The strain T1 showed resistance to penicillin which can be implied by an extensive resistance toward penicillin G in the *Lactobacillus* strain owing to defective cell wall mechanism, drug transporters, and cell wall autolytic systems (64). Many *Lactobacilli* are reported to be resistant to aminoglycosides (amikacin, streptomycin, etc.), glycopeptides like vancomycin, nucleic acid synthesis inhibitors (ciprofloxacin, norfloxacin, etc.) and to chloramphenicol, tetracycline, and erythromycin, as reported by Hana et al. (33). RS and T1 showed resistance to amikacin (protein synthesis inhibitor) which may be due to enzymatic antibiotic modification. Even though the strains resist these antibiotics, they are susceptible to most clinical antibiotics associated with opportunistic infections. The transmissible resistance against glycopeptide antibiotic vancomycin is significant, being the last effective antibiotic in treating certain multidrug-resistant pathogens-mediated infections (65).

BLIS from RS showed potent antagonistic activity against the target pathogens. The potential bioactive compounds can be further purified from these fractions for various applications. Similar studies show BLIS from *Enterococcus faecium* strains could inhibit *Clostridium perfringens* biofilm formation (66). Studies have suggested that bacteriocins influence and regulate the human microbiome by eliminating pathogen invasion and colonization with effective modulation of their dynamic gene clusters and narrow range of activity (67). Henceforth, BLIS from RS could potentially complement and enhance the strain’s competitive exclusion ability against gut pathogens. It was further confirmed by determining the survival-based assays in the *C. elegans* infection model. BLIS from RS potentially increased the survival rate of the worms infected with *S. enterica* MW116733, confirming the antipathogenic potential of the strain. These findings are similar to the study identifying a novel bacteriocin for controlling the mastitis-causing pathogen *S. aureus* strain RF122 in dairy cows (68). However, with T1, the observations were not much evident. It could be explained by the poor antagonistic potential of gram-positive bacteriocins in inhibiting a few strains of gram-negative genera, as explained elsewhere (69). Further investigations need to be conducted to detect these peptides’ exact nature.

Adhesion mechanisms of probiotic strains uphold a wide range of specific factors involving noncovalent interactions, steric forces, mucins, and physiological forces expressed by bacteria in terms of pili and lipoteichoic acid (70). However, variability in terms of the adhesion ability of the strains has been reported in earlier studies in a strain-dependent manner. Additionally, several studies have established the involvement of probiotic-derived enzymes and proteins, including several classes of hydrolases and transglycosylases (71). We should show that our strain possessed significant adhesion ability to HT-29 cell lines, which is an important criterion for accessing a probiotic strain. Our earlier investigations have shown that both strains possessed considerable antagonistic potential against enteric pathogens by conventional spot assay (results not shown). To further establish these observations, we checked the anti-adherence potential of RS and T1 to replace the pathogenic strains in HT-29 cells. We could establish that the strains showed significant replacement ability against both pathogen models, indicated by a marked reduction in the growth of the enteric pathogens in the 50–60% range. However, their competitive binding ability to the pathogens was not demonstrated in the *in vitro* environment. Earlier studies have demonstrated similar properties where probiotic strains may show variability in their competitive or exclusion ability against pathogen models (36). These observations may be based on the multifactorial properties of probiotic strains in inhibiting pathogens, specifically regarding the secretion of metabolites or antimicrobial peptides or their immunomodulatory properties (72).

It is well established that probiotic bacterial strains can control host immune defense mechanisms by activating a series of mediators from colon epithelial cells. Our results showed that supplementation of RS to HT-29 cells modulated the expression of pro and anti-inflammatory cytokines. Earlier investigations have proved that treatment of HT-29 with *L. acidophilus* increased the gene expression levels of the anti-inflammatory cytokines IL-6 and TNF-α and the chemokines CCL2 and CCL20 in a time-dependent manner (73). Similarly, a decrease in protein expression of TNF-α and an enhanced mRNA level of IL-10 was observed in *L. brevis* and *L. pentosus*-treated HT-29 cell lines (74). In the same context, HT-29 cells treated with *L. kefiri* SGL 13 showed significant downregulation of IL-8 (75). Various studies have described the advantageous effects of probiotic strains on intestinal health and established the significant consequence of immunobiotics on intestine cytokine profiles. These observations predominantly established the enhancement of TNF-α, IFN-γ, and the key regulatory cytokine IL-10 as explained by Marranzino et al. (35). Our preliminary data on cytokine expression profile (IL-6, IL-8, and IL-10) corroborate similar positive immunomodulatory roles in other potential probiotic strains.

## 5. Conclusion

Our studies could establish an *L. fermentum* strain from fermented rice water as a potential probiotic with food-based applications. We could also isolate a potential *L. fermentum* from the lemon pickle, which could propagate similar observations. Strains were competent in inhibiting pathogen loads *in vitro*, demonstrated significant adherence potential to ECM and HT-29 cell lines, and positively regulated the pro and anti-inflammatory cytokines. The production of Bacteriocin-like inhibitory substances may predominantly influence the potential of RS in inhibiting enteric pathogens, evidenced in the *C. elegans* survival model. We could claim that these strains isolated from potentially less explored sources like ricewater could be a promising strain to unravel their potential in treating gut dysbiosis and related inflammation, especially in undernourished populations. However, the need for the hour is to understand better the complex interaction of the gut microbiome with the putative probiotic strains in space and time. It is particularly relevant to tropical countries like India, where the number of food-derived established probiotics is comparatively limited.

## GenBank accession numbers

Sequencing data employed in the study have been deposited in GenBank with the accession number MN410703 and MN410702 for the *Limosilactobacillus fermentum* strains. The 16S rRNA sequence of *S. enterica* used in this study was deposited in GenBank with accession number MW116733.

## Data availability statement

The raw data supporting the conclusions of this article will be made available by the authors, without undue reservation.

## Author contributions

VP, AM, PB, and AV conceived and designed the experiments. VP, AJ, SN, AS, MP, TS, RR, PS, and KA performed the experiments. VP and AM wrote the manuscript and conducted the analysis. BN and SP reviewed and edited the manuscript. All authors contributed to the article and approved the submitted version.

## Conflict of interest

The authors declare that the research was conducted in the absence of any commercial or financial relationships that could be construed as a potential conflict of interest.

## Publisher’s note

All claims expressed in this article are solely those of the authors and do not necessarily represent those of their affiliated organizations, or those of the publisher, the editors and the reviewers. Any product that may be evaluated in this article, or claim that may be made by its manufacturer, is not guaranteed or endorsed by the publisher.
