# Supplementary material for: Harnessing the probiotic properties and immunomodulatory effects of fermented food-derived Limosilactobacillus fermentum strains: implications for environmental enteropathy
Source: Front Nutr. 2023 Jun 5;10:1200926. doi: 10.3389/fnut.2023.1200926 (PMC10277634; doi:10.3389/fnut.2023.1200926)
Supplement: Supplementary file 1 [file Data_Sheet_1.docx]

**Harnessing the Probiotic Properties and Immunomodulatory Effects of Fermented Food-Derived *Limosilactobacillus fermentum* Strains: Implications for Environmental Enteropathy**

Vidhya Prakash, Aravind Madhavan, Archana Palillam Veedu, Pradeesh Babu, Abhirami Jothish, Sruthy S Nair, Alin Suhail S, Meera Prabhakar, Thasleema Sain, Raveena Rajan, Priyanka S, K. Abhinand, Bipin G Nair, Sanjay Pal*

*** Correspondence:** Sanjay Pal: spal05@gmail.com

| Strains isolated | Source for isolation |
| --- | --- |
| *L. fermentum* MN410703 (RS) | Fermented rice water |
| *L. fermentum* MN410702 (T1) | Lime Pickle |
| *Staphylococcus warneri* MW336185 (SW) | Fermented rice water |

**Supplementary Table 1** Strains Isolated from Fermented Foods

**Supplementary Table 2** List of oligonucleotide qPCR primers

| Sl no. | Names | Sequences (5’-3’) |
| --- | --- | --- |
| 1 | IL 6F | GGAGAAGATTCCAAAGATGT |
| 2 | IL 6R | GCATCTAGATTCTTTGCCTT |
| 3 | IL 10F | TTGCTCTTGCAAAACCAAAC |
| 4 | IL 10R | TGTCTCAGTTTCGTATCTTC |
| 7 | IL 8F | GTGCATAAAGACATACTCCA |
| 8 | IL 8R | AGCCCTCTTCAAAAACTTCT |

**Supplementary Table 3** Gelatinase activity of RS and T1

| Temperature and time of incubation | | Gelatinase activity of the strains | |  |
| --- | --- | --- | --- | --- |
|  |  | SM | RS | T1 |
| 37^o^C (48 h.) | **+** | | **-** | **-** |
| 42^o^C (48 h.) | **+** | | **-** | **-** |
| 25^o^C (72 h.) | **+** | | **-** | **-** |
| 10^o^C (10 d) | **+** | | **-** | **-** |

SM: *Serratia marcescens* (MTCC 97*)* was used as positive control **[**(-) sign indicates the absence of gelatinase activity].

**Supplementary Table 4** Antibiotic susceptibility of the strains

| Antibiotic | Concentration (mcg) | Strains | |
| --- | --- | --- | --- |
|  |  | T1 | RS |
| Ampicillin | 10 | S | S |
| Cefoxitin | 25 | S | S |
| Amoxycillin | 10 | S | S |
| Gentamicin | 10 | S | S |
| Penicillin | 10^a^ | R | S |
| Amikacin | 30 | R | R |
| Chloramphenicol | 30 | S | S |
| Streptomycin | 10 | R | S |
| Erythromycin | 15 | S | S |
| Tetracycline | 30 | S | S |
| Vancomycin | 30 | R | R |

(S- Sensitive, R- resistant, INT- intermediate); a: concentration in units

**
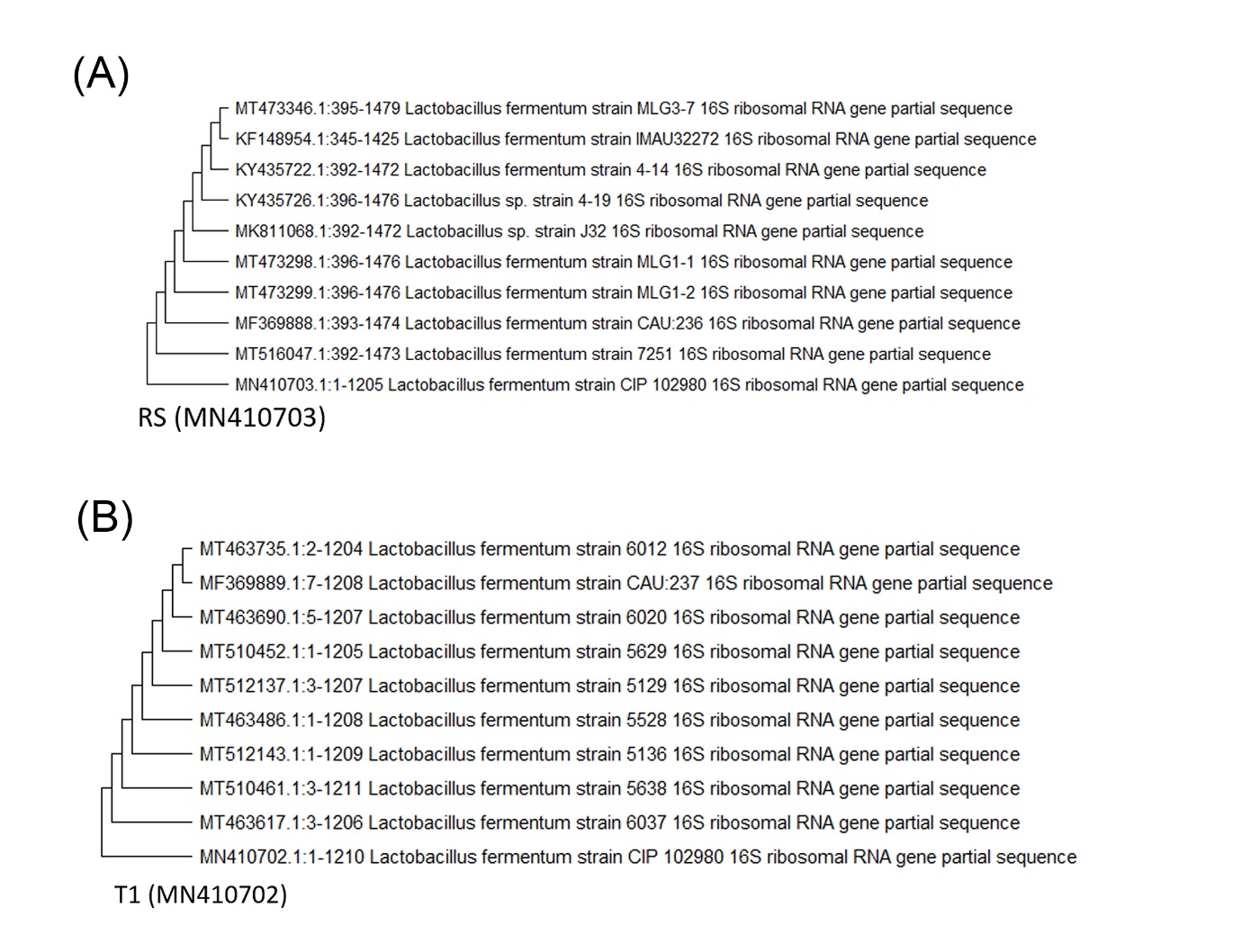
**

**Supplementary Figure 1** Phylogenetic analysis of 16S rRNA partial genome sequences of the RS (A) and T1(B). The Analysis was conducted with MEGA5 using neighbor-joining method. The optimal tree with the sum of branch length = 0.15357618 is shown. The tree is drawn to scale, with branch lengths in the same units as those of the evolutionary distances used to infer the phylogenetic tree. This Analysis involved 95 nucleotide sequences. All ambiguous positions were removed for each sequence pair (pairwise deletion option). There were a total of 1549 positions in the final dataset. Evolutionary analyses were conducted in MEGA X and top 10 hits are shown.


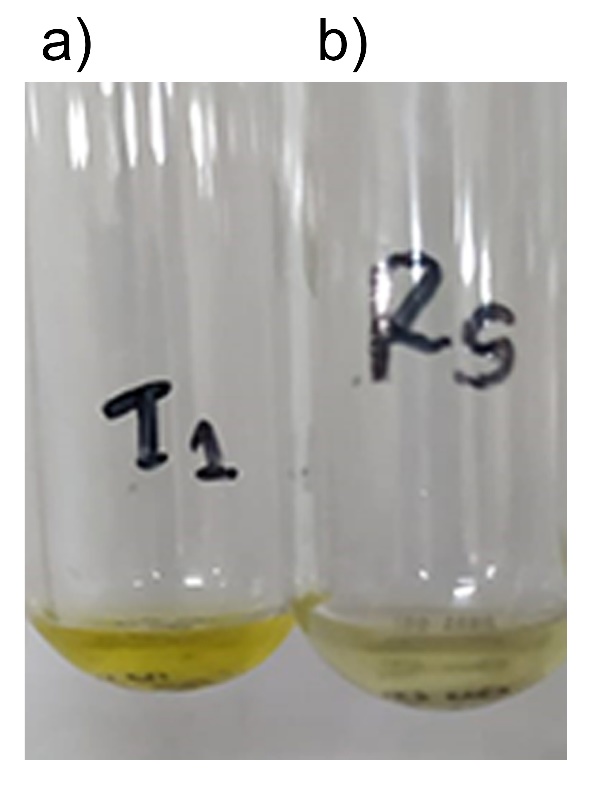


**Supplementary Figure 2:** Representation of ONPG Test For β-Galactosidase Production In T1 and RS.
